# EEG-based analysis of human driving performance in turning left and right using Hopfield neural network

**DOI:** 10.1186/2193-1801-2-662

**Published:** 2013-12-10

**Authors:** Mitra Taghizadeh-Sarabi, Kavous Salehzadeh Niksirat, Sohrab Khanmohammadi, Mohammadali Nazari

**Affiliations:** Department of Mechatronics Engineering, Tabriz Branch, Islamic Azad University, Tabriz, Iran; Department of Control Engineering, Science and Research Branch, Islamic Azad University, Tehran, Iran; Department of Control Engineering, Faculty of Electrical and Computer Engineering, University of Tabriz, Tabriz, Iran; Department of Psychology, University of Tabriz, Tabriz, Iran

**Keywords:** Driving, Fast Fourier Transforms (FFT), Hopfield network, Feature extraction

## Abstract

In this article a quantitative analysis was devised assessing driver’s cognition responses by exploring the neurobiological information underlying electroencephalographic (EEG) brain signals in a left and right turning experiment on simulator environment. Driving brain signals have been collected by a 19-channel electroencephalogram recording system. The driving pathway has been selected with no obstacles, a set of indicators are used to inform the subjects when they had to turn left or right by means of keyboard left and right arrows. Subsequently in order to remove artifacts, preprocessing is performed on data to achieve high accuracy. Features of signals are extracted by using Fast Fourier Transform (FFT). Absolute power of FFT is used as a basic feature. Scalar Feature selection method is applied to reduce feature dimension. Thereafter dimension-reduced features are fed to Hopfield Neural Network (HNN) recognizing different brain potentials stimulated by turning to left and right. The performances of HNN are evaluated by considering five conditions; before feature extraction, after feature extraction, before reduction of features, after analyzing reduced features and finally subject-wise Hopfield performances respectively. An increase occurred in each level and continued until it has reached its highest 97.6% of accuracy on last condition.

## Introduction

In recent decades human driving behavior has become one of the most interesting subjects and a large number of researchers have investigated it in different driving phases. In addition, physiological parameters such as EEG have been considered as a new performance measurement feature. Among the non-invasive techniques, brain activity can be inferred from EEG by placing electrodes on the surface of the scalp with millisecond resolution. The EEG is a well-documented technique which has the ability to characterize certain brain states in processing of different semantic categories (Hoenig et al. 2008; Pulvermuller et al. 1999; Kiefer 2001; Paz-Caballero et al. 2006; Proverbio et al. 2007; Fuggetta et al. 2009; Adorni and Proverbio 2009). The development of EEG-based interpreting approaches is an interesting application which makes real-time decoding systems possible (Muller et al. 2008). In order to decode the EEG-based tasks, three main aspects namely feature extraction (Sykacek et al. 2003; Ince et al. 2005; Wang et al. 2010), feature selection (Pregenzer and Pfurtscheller 1999; Garrett et al. 2003;Lal et al. 2004; Daly et al. 2011; Long et al. 2010) and classification approaches (Palaniappan et al. 2002; Peters et al. 2001) can be considered to analyze EEG signals (Coyle et al. 2005; Wolpaw et al. 2002; Coyle et al. 2006a;2006b). The EEG spectrum is normally composed of five different frequency bands: delta (1–4 Hz), theta (4–8 Hz), alpha (8–13 Hz), beta (13–30 Hz) and gamma (from 30 HZ).

Several studies relevant to current article such as characteristic of driving, drowsiness and fatigue detection have been evaluated by researchers. For instance, Shang et al. addressed to the driving characteristics analyzing cognitive state inside the brain. They used a relatively new method of multi-channel near-infrared spectroscopy (NIRS) to investigate the brain activation in a driving simulator by independently manipulating the cognitive demand. Left brain plays an initiative role, while right brain closely follows towards the activation degree of left brain, so they proved that there is a balanced tendency of symmetrical activation between left and right brain (Shang et al. 2007). Besides, Schier (2000) recorded EEG from four sites of scalp during both two-lap and replay driving tasks. Power spectra were computed to produce values of relative alpha activity and an increase was found in alpha activity as a result. Furthermore Chin-Teng et al. (2005a) suggested a system that combines EEG power spectra estimation, independent component analysis (ICA) and fuzzy neural network models to estimate drivers’ cognitive state in a dynamic virtual reality based environment. Also a relationship between driver’s style and driver’s ERP response was investigated (Chin-Teng et al. 2006). Power spectrum of ICA components and correlation between them was analyzed and Drivers were classified to aggressive or gentle based on the observed ERP difference. In addition EEG dynamics were studied in response to distraction during driving by event simulation, including unexpected car deviations and mathematics equations. Changes of EEG power spectra were measured and used to evaluate the brain dynamics in time and frequency domains (Chin-Teng et al. 2008).

Regarding investigation of drowsiness and fatigue in traffic accident, numerous physiological indicators are available to describe an individual’s level of alertness. The EEG signal has been shown to be one of the most predictive and reliable signals, since it is a direct measure of brain activity. An EEG based drowsiness-estimation system was developed using driver’s error, which is defined as deviations between the center of the vehicle and the center of the lane in the lane-keeping driving task (Chin-Teng et al. 2005b). Besides, a method was proposed that combine the EEG power spectrum, correlation analysis, principal component analysis, and linear regression models, to indirectly estimate the driver’s drowsiness level in a virtual-reality-based simulator (Liang et al. 2005). Additionally Papadelis et al. (2006) developed a method in order to prevent driving accidents and errors. They collected multichannel EEG data from 20 sleep-deprived subjects in real environmental conditions of driving. Observations of results show that an increase was appeared in slowing activity and an acute increase of the alpha waves, 5 to 10 seconds before driving events. In another relevant study, Nikhil et al. (2008) proposed a method to detect departure from alertness. They showed that the EEG power in the alpha and theta bands is highly correlated with changes in the subject’s cognitive state with respect to drowsiness through driving performance.

Recently artificial intelligence has become a convenient method for classification and prediction of biological investigations. For example, a quantitative analysis for assessing driver’s cognitive responses was devised by investigating the neurobiological information underlying EEG brain dynamics in traffic-light experiments. Event related potential features were then feed to a self-constructing neural fuzzy inference network (SONFIN) to recognize different brain potentials stimulated by red/green/yellow traffic events (Chin-Teng et al. 2007). Furthermore Hopfield neural network have been widely used in manifold fields such as regularized image restoration (Paik and Katsaggelos 1992), analogue computations of spiking neurons (Maassy and Natschlagerz 1997) and decomposing mixed pixels on images (Mei et al. 2010), However neither was applied on EEG pattern classification nor any biological signal. Due to Hopfield well-renowned in handwritings patterns recognition performances it can be a suitable choice of current article; which addresses Hopfield neural network recognition performances of driving signals. In this study, EEG signals were recorded in order to investigate human driving performance during right and left turning.

The purpose of this paper is to assess driver’s cognition responses in a left and right turning experiment on simulator environment using EEG, and it has attempted to classify left and right EEG brain signals. To perform EEG pattern classification, the rest of the paper is organized as follows: in section “Methods”, participants, experimental task, EEG recording system and preprocessing of EEG signal were explained. A sequence of approaches has been described namely: features extraction and feature selection (reducing the feature set dimensionality through selecting a subset of features) and Hopfield neural network in “Methods” section. Finally, the selected features related to EEG patterns were classified using Hopfield neural network. The complete details were expressed and discussed in “Results” and “Discussion and Conclusion” sections.

## Methods

### Participants

A total of ten adult volunteers (8 males and 2 females, age range: 18–28, mean age: 23 ± 3.4 SD) participated in current study. All participants were right-handed except one. They did not suffer from any psychological or neurological disorders and they had normal vision. All subjects were informed about the task prior to experiments. The study is conformed by the ethical guidelines of PAARAND specialized center.

### Experimental task

**Figure 1:**
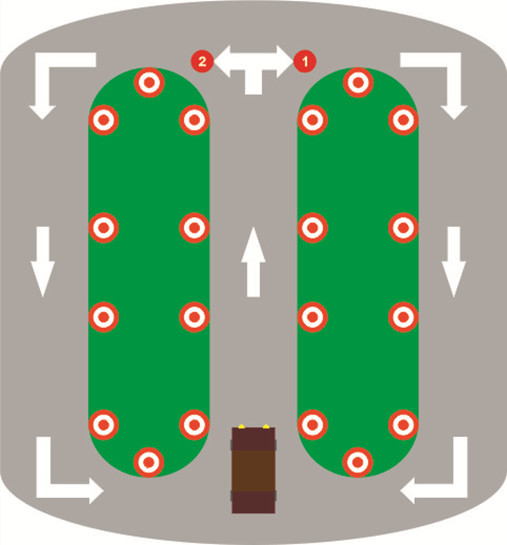
**Driving path.**

**Figure 2:**
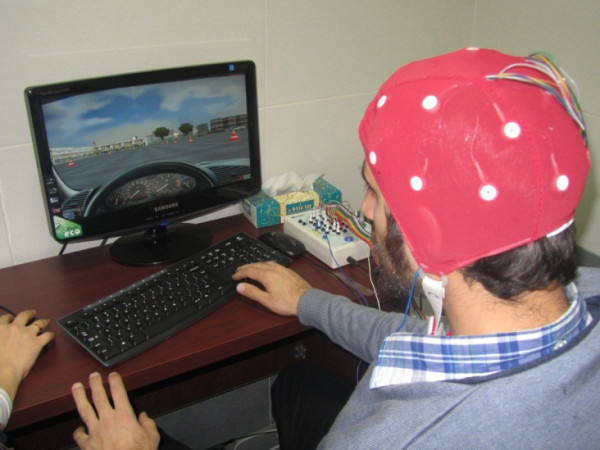
**Experimental task, one of participants during driving at 3D-driving school simulator.**

**Figure 3:**
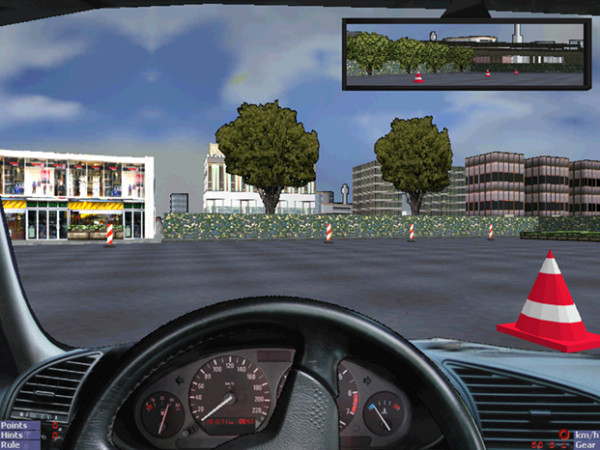
**3D-driving school simulator (copyright by BESIER 3D-EDUTAINMENT 2003).**

The simulator technique allows subjects to interact directly with driving task without risk of operating on actual machine. For this reason participants performed a driving task in a simulator environment. They were asked to drive along a pathway with indicators of turning to left and right. The path was like a butterfly wings (Figure 1) with no obstacles. Driving was comprised of four laps with four turns to right and four to left in each lap. Therefore each subject had experience of 32 turnings, 16 turnings to left and 16 to right. The task lasted 5 minutes and participants drove on cruise control mode with constant speed of 30 (km/h). Visual driving environment was implemented on the 17-inch LCD with 1280*800 resolutions (Figure 2). The specification for computer is described as follows: Pentium 4, 2.8 GHZ CPU, 1GB RAM, Windows XP professional and driving simulator program. The driving simulator was 3D-Driving School Simulator (copyright by BESIER 3D-EDUTAINMENT 2003) based on virtual environment (Figure 3).

### EEG recording system

Volunteers were fitted with a 19-channel electrode cap and prepared for EEG recording according to standard techniques. Recorded channels (FP1, FP2, F3, F4, C3, C4, P3, P4, F7, F8, T3, T4, T5, T6, FZ, CZ, PZ, O1, and O2) were selected from the international 10–20 set of electrode positions, with linked-ears montage (Miller et al. 1991). (The MCN system (Modified Combinatorial Nomenclature) renames four points of the 10–20 system T3, T4, T5 and T6 as T7, T8, P7 and P8, respectively). Subjects performed the experiment in a sound-dampened, electrically shielded booth. EEG signals were amplified with MITSAR hardware, and then sent through an analog- to- digital converter. Signals recorded at 500 Hz on a PC running digitize.

### Preprocessing

Due to the most dominant frequency bands of brain, (Delta (1–3.5 Hz), Theta (4–7.5 Hz), Alpha (8-14 Hz) and Beta (15-30 Hz)), a band-pass filter of 1-30 Hz transmitted over signals throughout (Michail et al. 2008). A 1-30 Hz phase-shift free Butterworth band-pass filter (12 dB/Octave) was used. Moreover two amplitude thresholds were considered; slow waves up to maximum 50 microvolts and ultimate 30 microvolts for fast waves. (Threshold values were chosen based on Alpha and Beta brain waves normal amplitudes (Sanei and Chambers 2007)). In order to correct detailed artifacts, independent component analysis (ICA) method was implemented. The “Infomax” algorithm was implemented in WinEEG software to analysis raw EEG signals (Delorme et al. 2007). Eye blink artifacts and some other artifacts were corrected using ICA method even if the EOG (Electrooculography) signal was not recorded (Hori and Cao 2011). This method is based on blind source separation procedure from multi-channel EEG data and spatial filtering of some components of EEG signal. After the decomposition of multi-channel signal, the components of signal related to artifacts were selected manually analyzing topographies and waveforms of components. In general the main components are horizontal and vertical eye movements besides temporal muscular activity, which all were predefined to the WinEEG software. The noisy components were selected and ICA algorithm was applied to the whole EEG data. In this study we had nineteen ICA components. However, actually up to two or three components were used maximally related to individuals.

### Feature extraction

Quantitative EEG analysis (QEEG) refers to extract features from EEG signal. Multi-channel EEG is digitized further adjusted to remove extra cerebral artifact, and subjected to spectral analysis using the fast Fourier transform (FFT). Extraction of features such as amount of absolute power at each electrode for each frequency band or as a function of frequency is carried out for individuals. All features used in this article were extracted using NeuroGuide; a software of quantitative EEG analyzer.

### Feature selection

In order to reduce the number of features easily, all the decompositions were normalized and reshaped to a row vector. For selecting essential and proper features for classification, scalar feature selection method was implemented using *T*-test criteria which it ranked all features. *T*-test criterion returns the significance level (p-value) of the test. The p-value is the probability, under the null hypothesis, of observing a value as extreme as or more extreme than the test statistic.

Absolute value of the criterion was used to rank features. Absolute value means that how much a feature is significant to separate two classes. Features with high absolute value were chosen and others were rejected. The scalar feature selection method considers feature ranking between the two classes.

### Hopfield neural network

Hopfield is a network with fully connected *N* artificial neurons which update their activation values. The update of a neuron depends on the other neurons of the network and on itself. A neuron *i* will be influenced by another neuron *j* with a certain weight *w*_*ij*_, and a threshold value (Hopfield 1984). There is a weight *w*_*ji*_ associated to input *i*. The connection weight from neuron *i* to neuron *j*, is *w*_*ij*_. In general always two conditions are imposed on the weight matrix: symmetry (*w*_*ij*_ = *w*_*ji*_) and no self-connections (*w*_*ii*_ = 0). The network also has an output. The state of the output is maintained until the neuron is updated. The training method consists of a single calculation for each weight. In Hopfield network instead of ones and zeros, which it is used in the other networks so far, the inputs are −1 and +1 (the neuron threshold is zero). This has to be true for the network to work correctly. Each weight is labeled by giving it a subscript showing which input it’s coming from and which neuron it’s going too. *w*_*ij*_ comes from input i and is going to neuron j. The training method is to multiply the value of each feature in each pattern corresponding to the index of the weight, so for *w*_*ij*_ the value of feature *i* and feature *j* were multiplied together in each of the patterns. Then the result is added up.

The new activation value (state) of a neuron is computed, in discrete time, by the function (1):12

Where *X* is the activation value of the *n* neurons, *W* is the weight matrix and *T* is the threshold of each neuron:345

The sign function is defined as:6

Hopfield network converges to a local state. The energy function of a Hopfield network in a certain state is (7):7

E1 is a general energy function. More often, E2 is used which is equivalent to E1.

The way Hopfield networks act, as a pattern is entered to the network, the Hopfield subject to a number of iterations updating all or part of the nodes to a specific value and stopped. The network neurons are then read out to see which pattern is in the network. The idea behind the Hopfield network is that patterns are stored in the weight matrix. The input must contain part of these patterns. The dynamics of the network then retrieve the patterns stored in the weight matrix. This is called Content Addressable Memory (CAM). The network can also be used for auto-association. The stored patterns in the network are divided in two parts: cue and association. By entering the cue into the network, the entire pattern, which is stored in the weight matrix, is retrieved.

## Results

Recorded EEG signals of 10 volunteers during simulated driving task were classified and various performances were achieved using Hopfield network. To clarify the effect of methods, firstly the result of time domain classification was explained; however the next section includes Hopfield performances in frequency domain by means of FFT feature extraction. Consequently feature selection method was applied on features. Then evaluation on the reduced features became more detailed; dividing participants’ features into three groups. Final assessment was performed subject wise in order to remove inter-subject-variability drawback.

### Time domain classification

As a first try to classify driving brain signals, 50 seconds of whole driving task is selected for turning left and the same for turning right per subject (the whole task was lasted 5 minutes, 90 seconds of which belongs to left turning, 90 seconds for right turning and 120 seconds for straight driving). The 50 seconds of the purest and noiseless part of signals were selected. Due to the 500 Hz sampling frequency of EEG recording system with 19 electrodes, selected signals were converted to a matrix size of 19 × 25000. It would be difficult to evaluate the matrix, pro oblong samples; therefore mean average of each electrode was computed and 20 matrixes with size of 19 × 1 were generated. Consequently the Matrix was normalized and then scaled between −1 and +1. Positive values were set to +1 and negative values were set to −1. In order to train Hopfield neural network, stable points and number of neurons were adjusted to 2 (two classes right and left turning) and 9 (the number of effective electrodes) respectively. Effective neurons were obtained by removing redundant channels which contains same value in both states. On the other word generally in current paper 19 neurons are available however in this case 9 of which has different value in two classes of right and left turning, the rest 10 neurons did not take into account because they had same value and no effect on network performance. This rule was applied to all parts of article and it will not be explained in following sections. Eventually Hopfield network was tested with 20 samples (10 samples for right and 10 for left). The Hopfield performance of 14.7% was obtained; too weak result!

### Frequency domain classification using FFT

In second analysis, frequency domain features were considered to classify signal patterns. In this part feature extraction was implemented on EEG data using Fast Fourier Transform. FFT was executed by NeuroGuide software. It transferred data to the absolute power of five major frequency domains namely delta, theta, alpha, beta and high beta ranged 1–3.5 Hz, 4–7.5 Hz, 8–12 Hz, 12.5–25 Hz and 25.5–30 Hz respectively. Consequently a data with size of 19 × 5 was formed per subject (19 channels × 5 frequency bands). At this stage five Hopfield networks were trained according to five frequency bands. Each network has two stable points with 19 electrodes in start, however only operative electrodes were considered to reduce neural network processing time and redundancy. Table 1 illustrates both the number of effective neurons and Hopfield network results tested by 20 patterns.

**Table 1:** **Classification without feature reduction**

Band name	Frequency range	Number of effective neurons	Network performance
Delta	1–3.5 Hz	7	25%
Theta	4–7.5 Hz	8	20.1%
Alpha	8–12 Hz	8	20%
Beta	12.5–25 Hz	11	10.4%
High Beta	25.5–30 Hz	6	5.6%

### Effect of feature selection

Earlier outcomes of Table 1 indicate low network performance. The reason should be investigated; hence feature selection and feature reduction may modify the results. Scalar Feature selection method with rule of ranking features was executed on data using MATLAB. Ranking features procedure is capable to rank features between two groups. In this paper there are two groups of left and right turnings. Types of features in this study are channels (electrodes) and frequency bands. The features with the most detached ability should be selected between two classes. Statistical criteria of *t*-test were implemented on extracted features for each subject individually. For more clarity, features were ranked based on absolute value related to *t*-test criterion which means that how much a feature is significant to separate two classes. Features with high absolute value were chosen and others were rejected. Seven electrodes names T5, Fp1, P3, O1, F7, T4 and Fp2 were picked among 19 electrodes; on the other hand another ranking was put in to practice in order to settle the most key frequencies of brain which delta and alpha had high ranking rate among five major bands. Finally 14 features (7 selected channels × 2 frequency bands) were chosen among 95 (19 channels × 5 frequency bands). The 14 prevalent features were classified by 14 neurons and 2 stable points by Hopfield network. Network performance of 42.1% was achieved by testing 20 patterns, which it is better than earlier results however it is not still a remarkable outcome. In the following section, 14 extracted features are evaluated in details.

### Subject’s group classification

**Figure 4:**
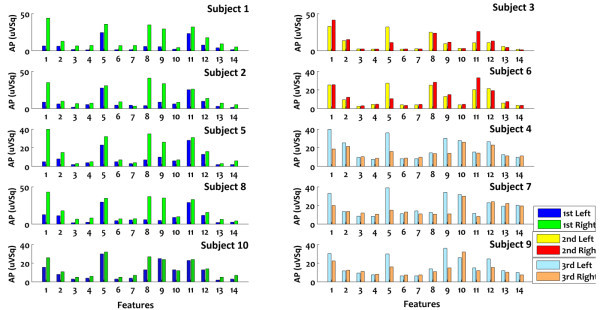
**Vertical axis is FFT absolute power (uv2) and horizontal axis is 14 features; Left side bar charts (dark blue and green) are behavior of first-group subjects (right-handed men), first and eighth FFT absolute powers of right-turning are much more than left turning.** Two up right bar charts (yellow and red) are behavior of second-group subjects (women), eleventh FFT absolute power of right-turning is more than left-turning and fifth FFT absolute power of left-turning is more than right-turning. Three down right bar charts (light blue and orange) are behavior of third-group subjects (people without driver’s license), first, fifth and ninth FFT absolute powers are more in left-turning than right-turning.

Listed 14 features in the previous part are bar graphed to investigate the behavior of individual characteristics of each participant. Table 2 indicates relevant feature numbers. Ten subjects’ bar graphs in Figure 4 illustrate the behavior of features per subject. Vertical axis is FFT absolute power (uVSq) and horizontal axis indicates features. By carefully studying the characteristics, three participant groups came into view and interestingly include: right-handed men, women and people without driver’s license; the subjects numbers 1,2,5,8 and 10 are in the first group. Third and sixth participants are included in second group, and the remained subjects 4, 7 and 9 form the third group. Common behavior in first group is that FFT absolute power of first and eighth features in right turning are much more than left turning. Joint behavior in second group is that eleventh feature in right turning is more than left turning but fifth feature acts in reverse manner. Finally in last group FFT absolute powers of first, fifth and ninth features are more in left turning than right turning (See Figure 4 for more detail). Consequently features with significant differences were selected to get better grouping. Finally Hopfield was designed with six stable points (three group × two driving states) and six neurons due to six selected features. The mentioned patterns are as following:8910111213

**Table 2:** **Features name is constructed of bands and channels both**

No.	1	2	3	4	5	6	7	8	9	10	11	12	13	14
Name	2d	15d	15a	11a	7d	1a	2a	9d	14d	7a	11d	1d	9a	14a
Band	delta	delta	alpha	alpha	Delta	alpha	alpha	delta	delta	alpha	delta	delta	alpha	alpha
Channel	FP2	T5	T5	F7	P3	FP1	FP2	O1	T4	P3	F7	FP1	O1	T4

For example 15d means that fifteenth electrode (T5) in delta band which it is summarized to only d.

The weight matrix is calculated based on pattern:14

**Figure 5:**
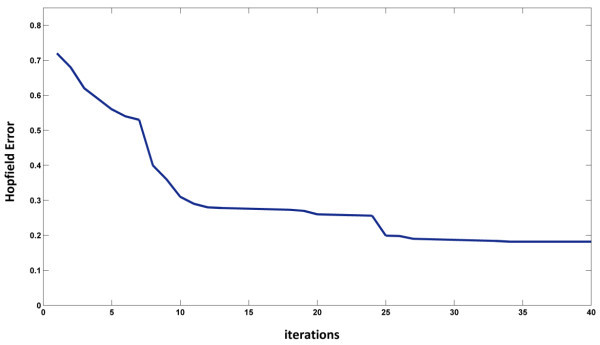
**Hopfield network error after 40 iterations with 0.182 mean error.**

To evaluate a Hopfield network which is well-known to recurrent associated memory network, 15% distortion was implemented on training data to test the Hopfield performance. Hopfield network should be reconstructing a pattern from a corrupted original. 15% distortion randomly implemented on training data 20 times. The average performance of 81.8% was achieved (p < 0.003). It is remarkable result (see Figure 5). This means that the network has been able to store the correct (uncorrupted) pattern in other words it has a memory. Finally notice that increasing distortion was lead to decreasing network performance.

### Individual Hopfield classification performance

Previous results predict that inter-subject variability could have negatively biased the classification performance. A fairer methodology was proposed in final part of result to obtain individual classification performance and from that to estimate the average performance of individual subjects. According to ten participants, ten Hopfield networks were designed separately relevant to each subject. Considering 16 turnings to right and 16 turnings to left for each subject, 10 turnings were selected to left and the same for right (This selection is according to artifact free signals that should be same for all subjects to have similar probability). Consequently Fourier transform was applied to each turning in order to extract features. This time we have separated train and test data to evaluate performance. 70% of data is used as training and the next 30% for testing. This means that Hopfield neural network was designed only based on training data without interference of test data. The extracted features of training data were reduced dimensionality and after full evaluation, Hopfield network was designed based on selected features. The process was repeated to 10 participants. Finally Hopfield networks were tested using remained 30% test data, and average of 97.6% performance was obtained (p < 0.001).

For statistically significant classification, random sub-sampling (Monte Carlo cross validation) were used. This method randomly splits the dataset into training and test data. Each time training and testing raw data were selected randomly to achieve different performance. The mentioned process was repeated 10 times and final performance was averaged and reported. The two-way parametric ANOVA test was used for evaluation over normality-checked performances and p-values are obtained for all subjects. Table 3 shows all details of results obtained by the last part study.

**Table 3:** **Performances of each subject’s Hopfield network with significant factor (p < 0.001)**

Subject	Performance
1	99.5%
2	96.4%
3	97.3%
4	94.6%
5	98.4%
6	97.6%
7	98.8%
8	97.9%
9	98.5%
10	96.9%

## Discussion and conclusion

**Figure 6:**
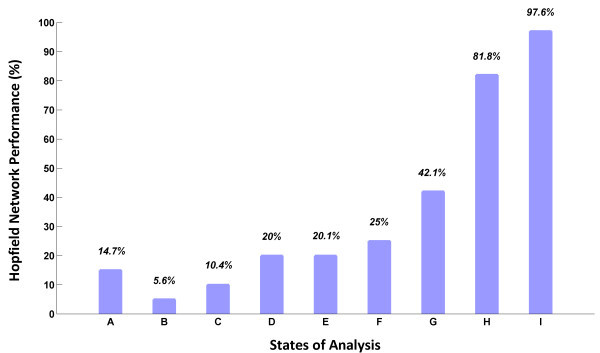
**Performance of study improvement due to using feature extraction, rankfeatures and analyzing selected features.** **(A)** Time domain classification, **(B)** High beta frequency band Classification, **(C)** Beta frequency band Classification, **(D)** Alpha frequency band Classification, **(E)** Theta frequency band Classification, **(F)** Delta frequency band Classification, **(G)** Classification after feature selection, **(H)** Subject’s group classification, **(I)** Individual classification performance.

The Electroencephalographic (EEG) signals were recorded for 10 participants, during the performance of driving task turning to right and left. Driving data were collected by 19-channel QEEG Mitsar/WinEEG based on 3D driving school simulator virtual environment. This work was focused on preprocessing and processing units. The statistical software-based ICA and filtering methods were implemented in order to remove eye-movement and eye blinks. Classification was done in several parts; a huge increase was appeared in the last evaluation. Features were extracted by FFT, ranked and reduced by *t*-test criteria. Hopfield neural network was reached to average performance of 97.6%. Figure 6 shows all network performances of study. Previous studies mainly focused on driver drowsiness or alertness prediction (Chin-Teng et al. 2005a;2005b; Liang et al. 2005; Papadelis et al. 2006; Nikhil et al. 2008; Michail et al. 2008), and different issues such as ride comfort (Mitsukura et al. 2009), driving style (Chin-Teng et al. 2006), and maximum band activity (Schier 2000; Chin-Teng et al. 2008). The researchers used different types of preprocessing and analyzing methods, such as ICA, PCA, FA (Factor Analysis), neural networks and support vector machine. However, they did not mention any analysis of driving basic actions. Basic actions include turning left or right, breaking and accelerating. This paper has introduced a new procedure analyzing turning left and right during driving with constant speed in a pre-designed path. The main contribution of current study is application of Hopfield neural network to classify EEG signals, which have capability of classifying three groups of people only using six features. The disadvantage of this study is its weakness for classifying non-homogenous participants. It is better to select subjects all in same gender with same driving experience in future studies. Furthermore the most important application of this study can be introduced as brain computer interface for intelligent driver assistance. Especially from last part results of study, it is derived that any BCI systems in driving process can be train individually based on its operator characteristics. Consequently individual trained BCI devices can lead more secure and reliable vehicles in the future.

### Consent

Written informed consent was obtained for the publication of this report and any accompanying images.
